# V-ATPase subunit B plays essential roles in the molting process of the Chinese mitten crab, *Eriocheir sinensis*

**DOI:** 10.1242/bio.048926

**Published:** 2020-05-29

**Authors:** Xin Hou, Xiaowen Chen, He Yang, Wucheng Yue, Jun Wang, Hua Han, Chenghui Wang

**Affiliations:** 1 Key Laboratory of Freshwater Aquatic Genetic Resources, Ministry of Agriculture, Shanghai Ocean University, Shanghai, 201306, China; 2 National Demonstration Center for Experimental Fisheries Science Education, Shanghai Ocean University, Shanghai, 201306, China; 3 Shanghai Engineering Research Center of Aquaculture, Shanghai Ocean University, Shanghai, 201306, China; 4 Department of Pharmacy, School of Medicine, Tongji University, Shanghai, 200433, China

**Keywords:** Chinese mitten crab, V-ATPase, Molting regulation, RNA interference, Epicuticle formation

## Abstract

Vacuolar ATPase (V-ATPase) is a proton pump driven by ATP hydrolysis, and it plays an important role in numerous biological processes, such as protein degradation and synthesis, cell growth, and cell autophagy. The V-ATPase subunit B (*VATB*) is a conservative and regulatory subunit required for ATP hydrolysis during proton pumping. The *VATB* of *Eriocheir* *sinensis* (*EsVATB*), which includes an open reading frame (ORF) length of 1467 bp encoding 489 amino acids, was cloned to unveil the biological function of *VATB* during the molting process of crustaceans. Spatial and temporal expression profiles showed that *EsVATB* was highly expressed in the posterior gill accompanied with the highest osmotic pressure in the premolt (PrM) stage. Meanwhile, the highest expression level of *EsVATB* was identified in the hepatopancreas and heart during the postmolt stage and epidermis in the intermolt stage, indicating that *EsVATB* may perform diverse biological functions in different tissues during the molting process. The individual crabs in the interference group showed a high mortality rate (74%) and a low molting rate (26%) and failed to form a new epicuticle in the PrM stage. Meanwhile, a significant difference in osmotic pressure was identified between the interference and control groups. Our results indicate that *EsVATB* is an indispensable functional gene that may participate in osmoregulation and help with the new epicuticle formation during the molting process of *E. sinensis*.

## Abstract (precis)

**Summary:** V-ATPase subunit B may participate in osmoregulation and help with the new epicuticle formation during the molting process of Chinese mitten crab *Eriocheir sinensis*.

## INTRODUCTION

Vacuolar ATPase (V-ATPase) is one of the major classes of ATPase in eukaryotes, coupling the energy released from ATP hydrolysis to proton transport (Forgac, 1989; Harvey, 1992; Wieczorek et al., 1999). V-ATPase is essential in regulating physiological processes, such as endocytosis, protein degradation, amino acid transportation, uptake of neurotransmitters, intracellular pH homeostasis and waste disposal in the cell through numerous cell signaling pathways (Forgac, 1989; Harvey, 1992; McGuire et al., 2017; Zhao et al., 2015). V-ATPase could modulate cell growth, death and proliferation through cell signaling pathways, such as the mammalian target of rapamycin (mTOR), transforming growth factor-β (TGF-β), and wingless (Wnt)/β-catenin signaling pathways (Cao et al., 2012; Cruciat et al., 2010; Fischer et al., 2012; Kim and Guan, 2015; Liron and Sabatini, 2014). V-ATPase is required for amino acid transportation to activate the mTOR pathway and promotes epithelial-mesenchymal transition through the TGF-β pathway (Cao et al., 2012; Zoncu et al., 2011). V-ATPase depletion affects the mitochondrial ATPase function in trypanosomes (Baker et al., 2015); mutations in V-ATPase caused impaired acidification in early endosomes in *Drosophila* (Vaccari et al., 2010). Loss-of-function of V-ATPase induces lysosomal deficiency and leads to Parkinson's disease (Dehay et al., 2012). The structure of V-ATPase is conserved in invertebrate and vertebrate species, and studies have been conducted to elucidate its essential roles in biological processes in numerous organisms (McGuire et al., 2017).

In arthropods, molting is a typical biological characteristic that directly determines behavioral and physiological activities, such as metamorphosis, growth and regeneration (Huang et al., 2015; Wang et al., 2013). To date, numerous works on V-ATPase have been conducted on insect species in terms of molting and growth regulation. Silencing the V-ATPase gene by RNA interference in *Helicoverpa armigera* led to high larval mortality and low growth rate (Jin et al., 2017). V-ATPase interacts with protein kinase A to regulate V-ATPase holoenzyme assembly/disassembly, which plays essential roles in energy utilization during molting of *Manduca sexta*, *Aedes aegypti* and *Drosophila melanogaster* (McGuire et al., 2017; Voss et al., 2007; Wieczorek et al., 2009). RNA interference of V-ATPase caused molting defect and developmental abnormalities in *Periplaneta fuliginosa* (Sato et al., 2017). However, although V-ATPase is important, studies on molting regulation in crustaceans are scarce (Das et al., 2018; Nagasawa, 2012; Roegner et al., 2018).

V-ATPases comprise two complex domains, a peripheral domain (V1) with eight subunits (A–H) that hydrolyzes ATP and a membrane integral domain (V0) that translocates protons (Toei et al., 2010). The activated V-ATPase activity requires fully assembled V1 and V0 complexes. Different subunits in the V1 complex participate in diverse biological processes; V-ATPase subunit H is essential for the survival and molting of *Locusta migratoria manilensis* (Li and Xia, 2012). RNA interference on the V-ATPase subunits A and E caused significant larval mortality in Colorado potato beetle and whiteflies (Baum et al., 2007; Upadhyay et al., 2011). Among the eight subunits, V-ATPase subunit (*VATB*), a conservative and indispensable regulatory subunit, is required for V-ATPase assembly and activity (Liu et al., 1996). Mutations in *VATB* cause renal tubular acidosis in humans (Karet et al., 1999). *VATB* was reported to be involved in tolerance to salt stress in wheat and in actin cytoskeleton remodeling in *Arabidopsis* (Ma et al., 2012; Wang et al., 2011). *VATB* is responsible for successful molting in *P. fuliginosa* (Sato et al., 2017). Moreover, *VATB* is widely studied as an osmoregulation gene in crustaceans; however, information about the function of *VATB* during molting in crustaceans is limited (Boudour-Boucheker et al., 2014; McNamara and Faria, 2012).

Chinese mitten crab *Eriocheir sinensis* is one of the most popular and economic aquacultured crustacean species in China (Wang et al., 2008). On the basis of the setal development characters of the second maxilla, the molting process of *E. sinensis* could be divided into three main stages, premolt (PrM), intermolt (InM) and postmolt (PoM) stage (Tian et al., 2013). Considerable research has focused on unveiling the molecular mechanism of molting in *E. sinensis* (Chen et al., 2017; Huang et al., 2015; Yuan et al., 2017). Numerous genes, such as the *ecdysone receptor*, *retinoid X receptor*, *molt-inhibiting hormone* and *crustacean hyperglycemic hormone*, which are involved in the ecdysone pathway, were studied to uncover the molting mechanism in *E. sinensis*. V-ATPase is a multifunctional proton pump that plays an essential role in diverse physiological processes, and the biological functions of *VATB* in molting regulation are largely undiscovered in crustaceans. Therefore, the role of *VATB* during the molting process in *E. sinensis* must be studied. In this study, the *VATB* of *E. sinensis* (*EsVATB*) was cloned, and its expression profiles were investigated in different tissues and molting stages during the molting cycle. Furthermore, RNA interference of *EsVATB* was conducted to demonstrate the biological functions of *EsVATB* in molting regulation in *E. sinensis*.

## RESULTS

### cDNA sequence of *EsVATB*

**Fig. 1.**
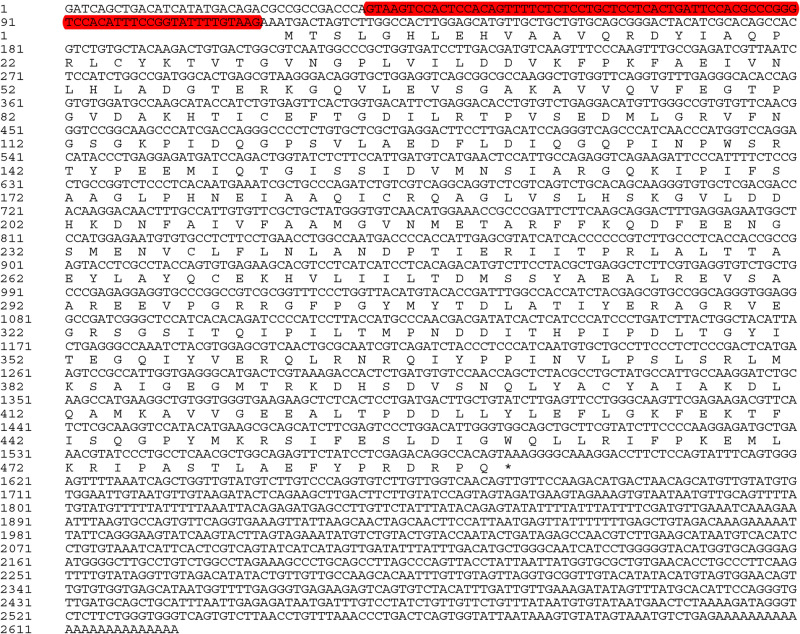
**cDNA sequence and predicted the amino acid sequence of *EsVATB*.** The shorter isoform is identical with the longer isoform except for the loss of 77 nucleotides at positions 38–114 (shown in red).

The complete cDNA sequence of *EsVATB*, with an open reading frame (ORF) length of 1467 bp encoding 489 amino acids, was obtained from posterior gill (G). Two isoforms with different 5′ untranslated regions (5′UTR) were identified in *EsVATB* (*EsVATB1* and *EsVATB2*). Both *EsVATB* isoforms were deposited in the GenBank database; the longer (*EsVATB1*) and shorter (*EsVATB2*) isoforms measure 2624 bp (GenBank Accession Number: MK389478) and 2547 bp in length (GenBank Accession Number: MK389479), respectively. Both isoforms share an identical ORF, but a 77 nucleotides insertion exists at positions 38–114 of the longer isoform in the 5′UTR region (Fig. 1).

**Fig. 2.**
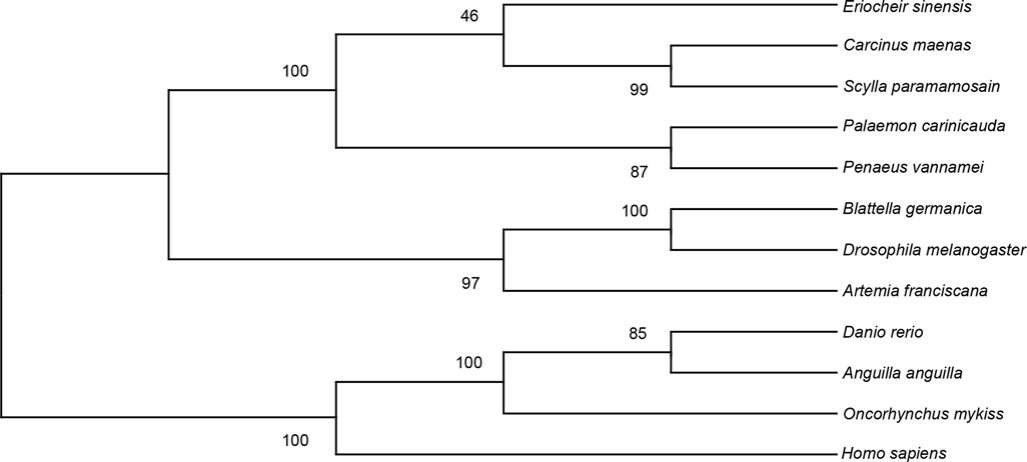
**Phylogenetic tree of *VATB* from selected species.** The tree was constructed using MEGA X with 1000 bootstrap values.

BLASTP search by the deduced amino acid sequence of *EsVATB* revealed the highly conserved structure of this protein compared with other species. Three well-conserved domains were identified, namely, ATP-synt_ab_N domain (28–94), ATP-synt_ab domain (151–378), and ATP-synt_ab_C domain (395–486) through SMART database search. The phylogenetic tree presented three distinct phylogenetic clades (Crustacean, Insecta, and Vertebrate) as outgroups. V-ATPase subunit B of *E. sinensis* was closely clustered with other two crab species, *Carcinus maenas* and *Scylla paramamosain*, in the crustacean clade, indicating the conserved function of *VATB* in crustaceans (Fig. 2).

### Expression of *EsVATB* in various tissues and molting stages

**Fig. 3.**
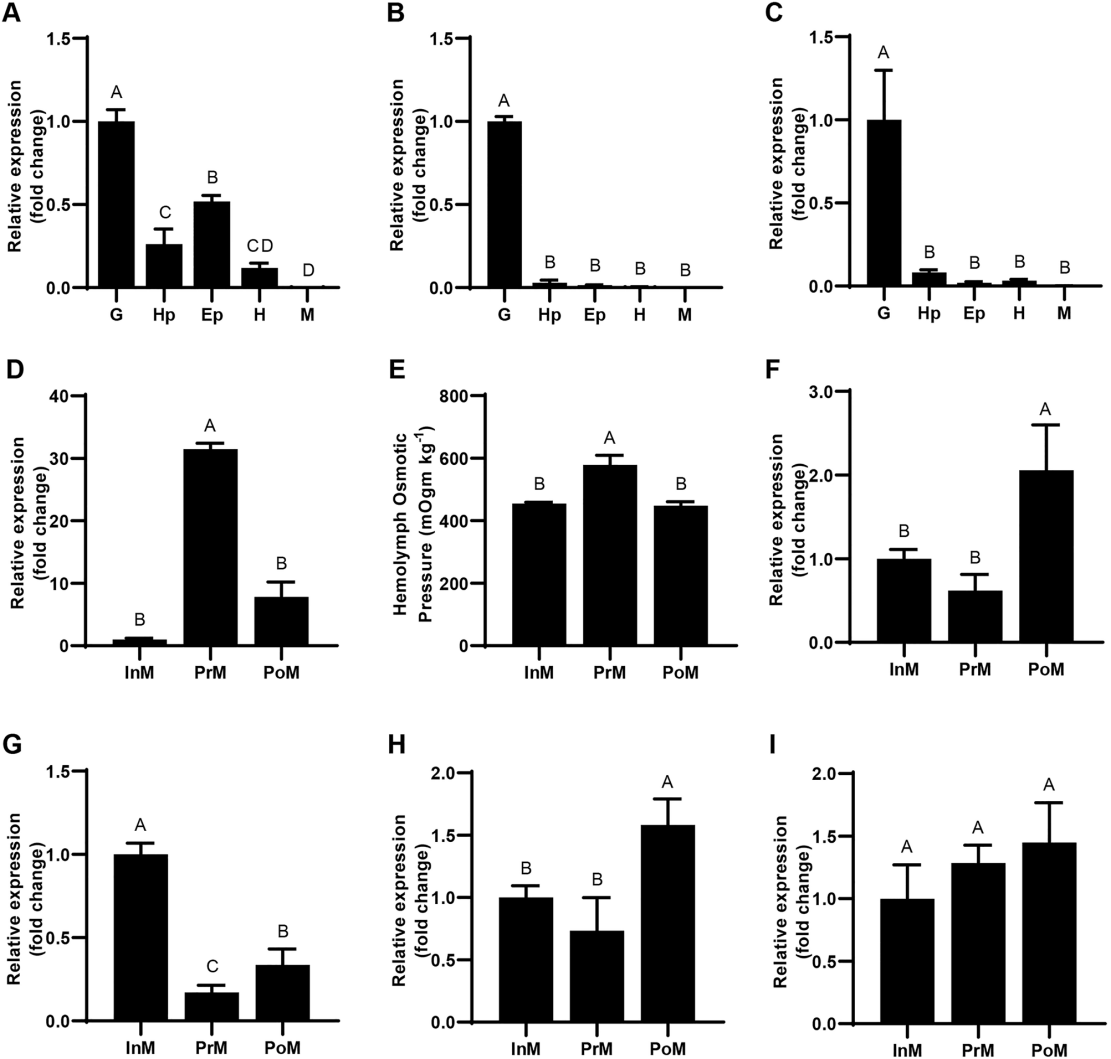
**Expression levels of *EsVATB* in different tissues and molting stages and the hemolymph osmotic pressure in different molting stages.** (A–C) Relative *EsVATB* expression levels in five tissues in InM, PrM and PoM, respectively. (D,F,G,H,I) Relative *EsVATB* expression levels in G, Hp, Ep, H and M, respectively, during molting. (E) The hemolymph osmotic pressure during molting. Bars that do not share a common letter (A,B,C and D) indicate highly significant differences among different tissues or stages (*P*<0.01).

*EsVATB* was expressed in all the studied tissues. However, the highest expression level was identified in the G tissue in the whole molting process (*P*<0.01) (Fig. 3A–C). Regarding each studied tissue in different molting stages, diverse expression patterns were identified for *EsVATB*. Regarding the G tissue, the expression level of *EsVATB* was the highest in the PrM stage (*P*<0.01) (Fig. 3D). Interestingly, the highest significant osmotic pressure level was also identified in the PrM stage, consistent with the expression level of *EsVATB* in the G tissue during the molting process (Fig. 3E). *EsVATB* was significantly highly expressed in hepatopancreas (Hp) and heart (H) in the PoM stage. Meanwhile, for epidermis (Ep), a significantly high expression of *EsVATB* was identified in the InM stage (*P*<0.01) (Fig. 3F–H). No significant expression difference in walking leg muscle (M) was identified in the different molting stages (*P*>0.05) (Fig. 3I).

### RNA interference of *EsVATB* during the molting process

**Fig. 4.**
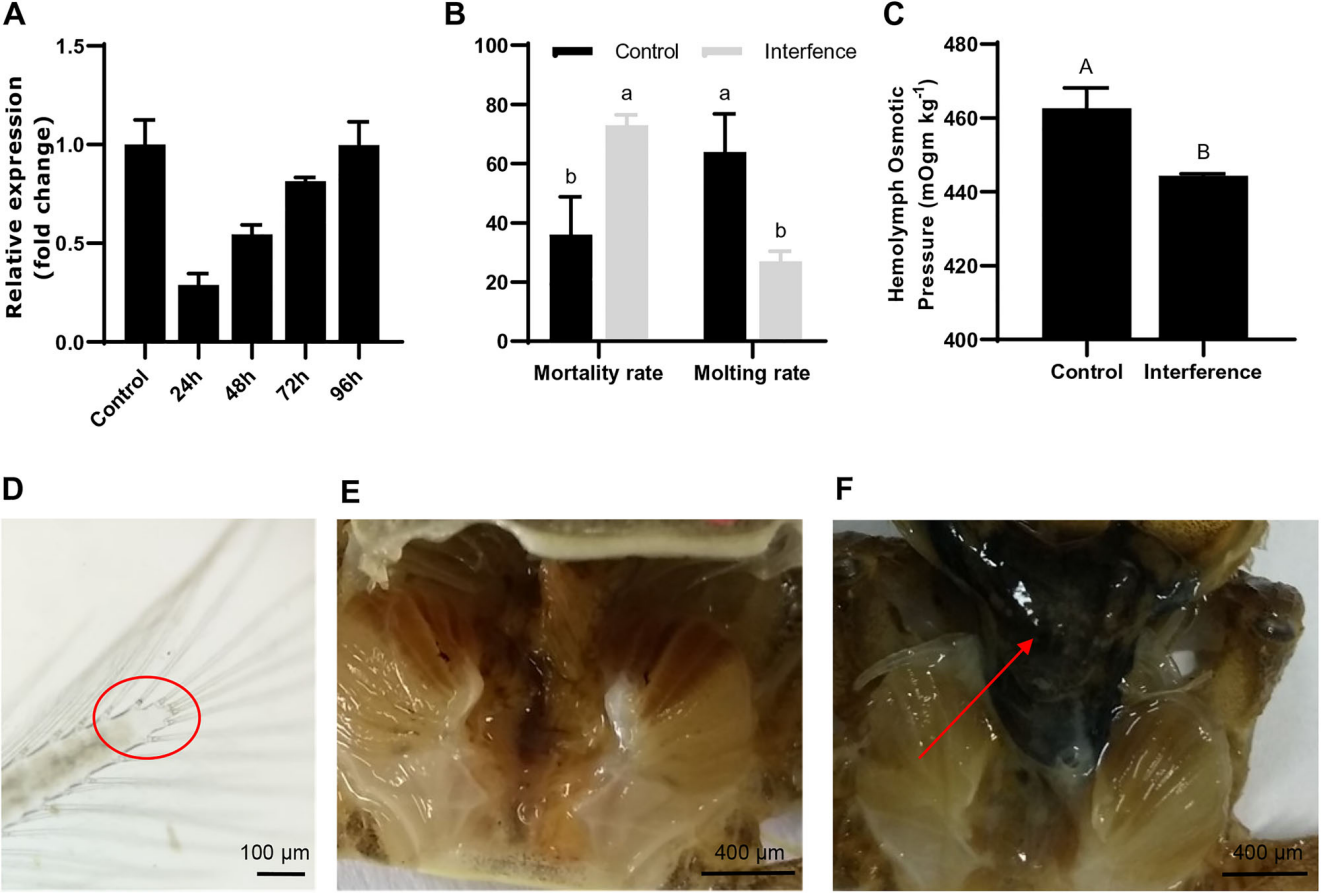
***EsVATB* dsRNA experiment during molting stages of *E. sinensis*.** (A) Efficiency of dsRNA-EsVATB injection revealed by qRT-PCR. (B) The final mortality and molting rates in the control and interference groups. (C) Hemolymph osmotic pressure of the control and interference groups after the fourth injection. (D) Cavities that appeared in the bristles of mandible of the crabs in the PrM stage. (E) Crabs in the interference group at the PrM stage with no new epicuticle formed. (F) Crabs in the control group at the PrM stage with a new epicuticle formed. Bars that do not share a common letter (A and B) indicate highly significant differences (*P*<0.01), (a and b) indicate significant differences (*P*<0.05).

The efficiency of *EsVATB* double-stranded RNA (dsRNA) was evaluated by quantitative real-time PCR (qRT-PCR) analysis. The expression level of *EsVATB* was reduced to 68% at 24 h, 46% at 48 h, and 19% at 72 h (Fig. 4A). No significantly reduced expression level of *EsVATB* was identified at 96 h after injection.

During the *EsVATB* dsRNA treatment, no significant morphological changes were identified between the interference and control groups until 20 days after the fifth injection, when dead crabs were first identified in the interference group. After the *EsVATB* dsRNA interference treatment, the mortality and molting rates reached 74% and 26% in the interference group, respectively. Meanwhile, the mortality and molting rates totaled 36% and 64% in the control group, respectively (Fig. 4B). After dissection and observation, the dead crab individuals in the interference group all remained in the PrM stage (Fig. 4D). However, the new epicuticle was not formed (Fig. 4E) compared with the control group with the completely formed new epicuticle (Fig. 4F). Meanwhile, the osmotic pressure of crabs in the interference group was significantly lower than that in the control group (Fig. 4C).

## DISCUSSION

In this study, we cloned the full-length cDNA encoding *EsVATB* and studied its essential roles in regulating the molting process of *E. sinensis*. Similar to the *VATB* gene in other species, the V-ATPase subunits B of *E. sinensis* also includes two isoforms, however, the two isoforms show identical ORFs with the alternatively spliced 5′UTR (Schredelseker and Pelster, 2010; Van et al., 1994; Weihrauch et al., 2001). The 5′UTR of genes plays indispensable roles in gene expression regulation after transcription, thus contributing to specific biological processes (Hughes, 2006). The two isoforms identified in this study may exhibit different expression regulation patterns in *E. sinensis*. However, further functional studies need to be conducted to confirm and unveil the related mechanism.

The tissue expression pattern of *EsVATB* is similar to that of other aquatic crustaceans, with the highest expression identified in the osmoregulatory organ, that is, the gills (Ali et al., 2017; Li et al., 2015). Crustacean gills are a multifunctional organ essential for osmotic and ionic homeostasis, except for their role in gas exchange in crustaceans (Freire et al., 2008). In this study, the expression level of *EsVATB* was consistent with the dynamic changes in osmotic pressure, which increased sharply in the PrM stage, indicating the essential roles of *EsVATB* in osmoregulation during molting (McNamara and Faria, 2012). During periodic molting of crustaceans, absorption of water will enlarge the cell volume before molting, which will unbalance the osmotic pressure in cells (Nako et al., 2018). The upregulated *EsVATB* expression level in the PrM stage of G tissue may indicate the fundamental roles of water absorption and osmoregulation during the molting process of *E. sinensis*. Furthermore, the extremely significant difference in the osmotic pressure between the interference and control groups indicates that *EsVATB* may participate in osmoregulation during molting.

A new epicuticle, which is vital for the whole molting process of crustaceans, is formed during molting stage and will become calcified afterward. In this study, during the whole *EsVATB* injection process, no significant morphological changes were initially identified between the interference and control groups. However, 20 days after the fifth injection, dead crabs were discovered in the interference group. On the contrary, at the same time (20 days after injection), a new epicuticle had generated in the control group, indicating that *EsVATB* RNA interference had impeded the new epicuticle formation in the interference group. Meanwhile, our qRT-PCR experiment also presented that *EsVATB* was highly expressed in the Ep in the InM stage, and this finding may strongly indicate the essential roles of *EsVATB* in new epicuticle formation during molting. V-ATPase is necessary for ATP hydrolysis and energy metabolism, and the new epicuticle formation defect may due to the abnormal ATP hydrolysis in *E. sinensis* caused by *EsVATB* RNA interference (McGuire et al., 2017; Toei et al., 2010). The RNA interference of *VATB* gene of *P. fuliginosa* caused similar phenomena, including molting defects and developmental abnormalities, which are consistent with our results (Sato et al., 2017). During molting, a series of tissue structures of *E. sinensis* had been degraded or digested to provide the required nutrition for molting. The internal old epicuticle was dissolved, and various substances, such as wall proteins, carbohydrates, collagen fibers, Ca^2^^+^ and Mg^2+^, would be reabsorbed to ensure complete molting (Tian et al., 2013). The expression and immunocytochemical analyses of the *VATB* gene of *Porcellio scaber* revealed that *VATB* is required for the formation and resorption of CaCO_3_ in the epithelial cells during mineralization and demineralization, which indicate that the RNA interference of *EsVATB* may hinder the reabsorption of the old exoskeleton before molting and eventually lead to incomplete molting (Andreas et al., 2004).

In summary, *EsVATB* is a conservative functional gene that plays essential roles in molting regulation of *E. sinensis*. The RNA interference of *EsVATB* may hinder osmotic pressure regulation and new epicuticle formation during molting, resulting in the attempted molting of *E. sinensis*. However, the molecular mechanism of *EsVATB* on molting regulation still needs further functional studies.

## MATERIALS AND METHODS

### Experimental animals and samples

Five-month-old crabs (around 3–4 g) were collected from the Genetic Resource Station of Shanghai Ocean University (Shanghai, China). All the collected crabs were cultured in a container with fresh circulating water at room temperature and fed twice daily. G, Hp, Ep, H and M from six crabs were sampled at each molting stage (PrM, InM and PoM stage) in accordance with the setal development characters of the second maxilla and were immediately stored at −80°C before RNA extraction. The sampling procedures were approved by the Institutional Animal Care and Use Committee of Shanghai Ocean University.

### RNA extraction and full-length cloning of *EsVATB*

RNA was extracted from 50–100 mg sampled tissues using Trizol reagent (Takara, Dalian, China) according to the manufacturer's instructions. The quantity and quality of RNA were examined by NanoDrop 2000 (Thermo Fisher Scientific, USA) and 1.0% agarose gels, respectively. cDNA was synthesized using a PrimeScript™ RT Reagent Kit (Takara, Dalian, China) according to the manufacturer's instructions. The RNA extracted from G tissue was used for full-length cDNA synthesis by SMARTer RACE 5′/3′ Kit (Takara, Japan). Table 1 shows the sequences of primers used for cloning *EsVATB*. The identified sequence was verified and analyzed using the National Center for Biotechnology Information (NCBI) BLAST tool (http://blast.ncbi.nlm.nih.gov/). SMART (http://smart.embl-heidelberg.de/) was used to predict conservative domains with the default parameters.

**Table 1.**
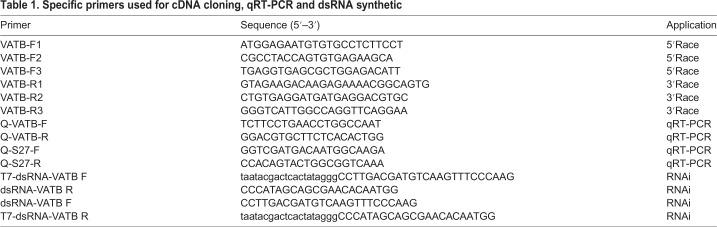
**Specific primers used for cDNA cloning, qRT-PCR and dsRNA synthetic**

### Phylogenetic tree construction

The gene sequences from *C. maenas* (AAF08281.1), *S. paramamosain* (ANC67975.1), *Palaemon carinicauda* (AHA11058.1), *Penaeus vannamei* (XP_027229418.1), *Blattella germanica* (AQU14352.1), *D. melanogaster* (CAA48034.1), *Artemia franciscana* (AAP37188.1), *Danio rerio* (NP_878298.1), *Anguilla anguilla* (AAD55091.1), *Oncorhynchus mykiss* (NP_001118069.1), and *Homo sapiens* (NP_001683.2) were download from the NCBI database to construct the *VATB* phylogenetic tree among insects and crustaceans. Sequence alignment was conducted by Clustal W software, and a phylogenetic tree was constructed using the MEGA X software with 1000 bootstrap values based on the maximum likelihood method (Tamura et al., 2013; Thompson et al., 1994).

### qRT-PCR

qRT-PCR was performed to determine the expression of *EsVATB* in different tissues and molting stages. qRT-PCR was conducted using SYBR Green Premix Ex *Taq* (Takara, Dalian, China) in a Rotor-Gene Q 2PLEX real-time PCR system (Qiagen, Germany). The total reaction mixture included 6.25 µl SYBR Green Premix Ex *Taq*, 0.25 µl of each sense and antisense primer (10 µM), 1 µl cDNA, and 4.25 µl ddH_2_O. The standard curve was first obtained using fivefold dilutions of the cDNA for *EsVATB* and the specific gene primer. The ubiquitin/ribosomal S27 fusion protein gene served as an internal reference gene (Huang et al., 2017). Three biological replicates and three technical replicates were obtained for the qRT-PCR. The expression results were presented as the means±standard error calculated using the 2^−ΔΔCt^ method (Livak and Schmittgen, 2001). The expression of *EsVATB* was measured in the G tissue and InM stage as internal calibration control in different tissues and molting stage experiments, respectively.

### Synthesis of *EsVATB* dsRNA and injection bioassay

Based on the ORF of *EsVATB*, the interference segment was designed by RNAi target online design software BLOCK-iT™ RNAi Designer (http://rnaidesigner.thermofisher.com), and the primers dsRNA-VATB F and dsRNA-VATB R were designed to amplify a 575 bp sequence fragment in the coding region of *EsVATB* (Table 1). Then, the primers T7-dsRNA-VATB F/dsRNA-VATB R and T7-dsRNA-VATB R/dsRNA-VATB F were used to add a T7 promoter to the *EsVATB* dsRNA synthesized segment. *EsVATB* dsRNA was amplified with RiboMaxTM Promega Large Scale RNA Production Systems-T7 Kit (Promega, USA) according to the manufacturer's instructions.

A total of 30 crabs (1±0.1 g) in the InM stage were divided into five groups to evaluate the efficiency of *EsVATB* dsRNA. Four groups were injected with *EsVATB* dsRNA using a microinjector (Sangon Biotech, China) from the proximal arthrodial membrane at the base of the fourth walking leg of each crab (4 µl, 1 µg µl^−1^). G tissue from each interference group were sampled at 24, 48, 72 and 96 h after injection. Diethyl pyrocarbonate (DEPC) H_2_O was injected using the same method in the control group, and G tissue were collected after 24 h. The expression level of *EsVATB* was detected by qRT-PCR experiment. After evaluation of the efficiency of *EsVATB* dsRNA, 30 crabs (1±0.1 g) at the same molting stage were collected (4 days after molting). These 30 crabs were randomly divided into two groups (interference and control groups with 15 crab individuals in each group). *EsVATB* dsRNA (4 µl, 1 µg µl^−1^) and an equal volume of DEPC H2O were injected into the interference and control groups, respectively. *EsVATB* dsRNA and DEPC H2O were continually injected in the interference and control groups every 4 days until the next molting. During the experiment, the molting and mortality information were observed and recorded every day.

### Measurement of hemolymph osmotic pressure

Crabs in different molting stages in the interference and control groups were selected to measure the hemolymph osmotic pressure. Sampling in different molting stages was based on structural changes. Sampling in RNAi experiment was performed after the fourth injection according to the previous research. Three biological replicates were employed. Approximately 400 µl hemolymph was extracted through the proximal arthrodial membrane at the base of the fourth walking leg of the crabs by a medical grade syringe. The hemolymph was recovered after centrifugation for 10 min in a microfuge, and the cell clot was removed before osmotic pressure measurement by a Micro-Osmometer (Löser, Germany).

### Statistical analysis

One-way ANOVA with Tukey's test was performed using SPSS 25.0 to determine any significant differences. Results were expressed as mean± standard deviation. Significance was accepted at the level of *P*<0.05.
